# On the Employment of Lactate of Iron

**Published:** 1841-01

**Authors:** 


*On the Employment of Lactate of Iron.* By MM. Gelis and Conte.—After stating some objections to the preparations of iron in common use, the authors give their reasons for supposing that the lactate of the protoxide of iron is superior to all other ferruginous preparations. These are that lactic acid is universally distributed throughout the body; and that all authors have endeavored to administer iron in the form most easily soluble in the gastric juice. Berzelius, Tiedemann and Gmelin, Dumas, Leuret and Lassaigne, have shown that the gastric juice has sufficient lactic acid to account for its dissolving property. They find that the most useful preparations of iron are those most soluble in lactic acid and the reverse, and therefore consider it probable that iron after administration is converted into lactate of iron. Thus they were led to give the lactate direct.

M. Bouillaud and other members of the Academy administered this preparation in twenty-one cases of anemia and chlorosis; but though they speak favorably of it, it did not appear to them to possess any decided advantage over other soluble preparations of iron.—*Bul. de l'Acad. Ib.*

